# An improved flow-through phototransducer

**DOI:** 10.1155/S1463924682000467

**Published:** 1982

**Authors:** T. J. Sly, D. Betteridge, D. Wibberley, D. G. Porter

**Affiliations:** 1 Department of Chemistry University College Swansea Singleton Park Swansea SA2 8PP UK; 2 Laboratory of the Government Chemist Cornwall House Stamford Street London SEI 9NQ UK; 3 B.P. Research Centre Sunbury-on-Thames TW16 7LN UK; 4 Department of Electronic Enfineering UMIST Manchester M60 1QD UK


Journal of Automatic Chemistry, Volume 4, Number 4 (October–December 1982), pages 186–189

### An improved flow-through photo-transducer

**T. J. Sly, D. Betteridge* and D. Wibberley†**

*Department of Chemistry, University College Swansea, Singleton Park, Swansea SA2 8PP, UK*

**and D. G. Porter**

*Laboratory of the Government Chemist, Cornwall House, Stamford Street, London SE1 9NQ*

### Introduction

In 1978, a flow-through phototransducer was developed in our laboratories at University College Swansea; it used a light-emitting diode (LED) as the light source and a silicon phototransistor as the detector [1]. A number of these units were constructed and have proved extremely useful as sensitive, low-cost detectors in flow-injection analysis (FIA).

Over a period of time, various flowcell designs of different path-length and diameter have been assessed and used in a number of applications. For each design it was found necessary to change the values of certain electronic components in the detector circuit in order to accommodate the different light levels incident upon the phototransistor as a result of the different path-lengths and areas, so that in practice it has proved difficult to try more than one type of flowcell for any particular application.

Problems of long-term reliability have been encountered with the external connections between the electronic circuit and the flowcells, which were vulnerable both to mechanical damage and to chemical attack. In addition, under certain conditions the external leads from the phototransistor are sensitive to electrical interference. The phototransducer was redesigned to overcome these deficiencies, and at the same time to improve the overall performance, as described in detail below.

### Design details

### Electronic circuit

The following points were considered in preparing the new design:

(1) The level of current flowing through the LED and the gain of the amplifier should be switchable to allow a wide variety of flowcell geometries to be accommodated without the need for modifications to the circuit.
(2) The circuit of the new unit should be constructed on a printed circuit board (PCB) to facilitate easy assembly.
(3) The flowcell should be accommodated inside the same housing as the circuit, preferably by mounting directly to the PCB, thus avoiding the problems of interference associated with external wiring.

The circuit diagram of the redesigned phototransducer is shown in figure 1. The LED is driven from a constant current source which ensures a stable light output, and the current level is switchable by means of S4. The phototransistor is biassed from a stable voltage source and the user has the option of selecting an external voltage source by means of S3 if required. The gain of the unit is defined by R5 and R6, and is switch-selectable. R5, C8 and R6, C9 are chosen so that the response of the unit rolls off above 1 Hz to prevent amplification of spurious transients. Finally, provision is made for the output to be attenuated by means of RV1 if required.

To zero the unit, the voltage at S1 is adjusted, either by varying RV2 or by applying a suitable external voltage, such that the current flowing in R7 is exactly equal to the current flowing through the phototransistor. At this point the net current flowing into the inverting input of IC1 is zero, resulting in zero output. When an absorbing species enters the flowcell, the intensity of light reaching the phototransistor falls, with a consequent decrease in the current flowing through it. There is now a net flow of current out of the inverting input of IC1, with the result that a positive voltage is produced at the output.

The revised circuit design made provision for the flowcell to be mounted onto a 24-pin DIL header and fitted into a similar DIL socket mounted directly on the PCB. The entire assembly, including the flowcell, was mounted on a PCB 45 mm × 105 mm in size, and housed in a small die-cast box. All external connections were made via a single nine-way 'D' connector mounted on the board.

### Flowcell

It was felt that it would be advantageous to devise a new type of flowcell, compatible with the new circuit, but more robust and simpler to produce than earlier types.

Eventually the cell shown in figure 2(*a*) was devised. It consisted of a glass capillary of 1 mm diameter fitted at right angles through the walls of a piece of small-bore opaque rubber or heat-shrinkable tubing. An LED and a phototransistor were fitted into the two ends of the tube, and the assembly was mounted onto the base of a small plastic plug-in module using epoxy resin adhesive, and coated in black paint to exclude stray light (figure 2 [*b*]). The cover was then fitted, after which the cell was encapsulated in epoxy resin.

The capillary was gripped tightly between the walls of the tube, thus ensuring that all light reaching the phototransistor had passed through the capillary, and preventing any leakage of light from the light source to the detector. In tests, this cell proved to be equally sensitive compared with other types of cell of similar path-length, whilst offering a considerable improvement in the resolution of the fine structure present in FIA peaks, due to the fact that the absorbance was measured at right angles to the direction of the flow.

The other advantages of this type of cell, which arose due to the non-intrusive nature of the sensors, were that (*a*) it did not disrupt the flow pattern of the liquid passing through it, since the

** Present address: B.P. Research Centre, Sunbury-on-Thames TW16 7LN, UK.*

*† Present address: Department of Electronic Engineering, UMIST, Manchester M60 1QD, UK.*

 0142-0453/82/0404 0186 $05·00 © 1982 Taylor & Francis Ltd

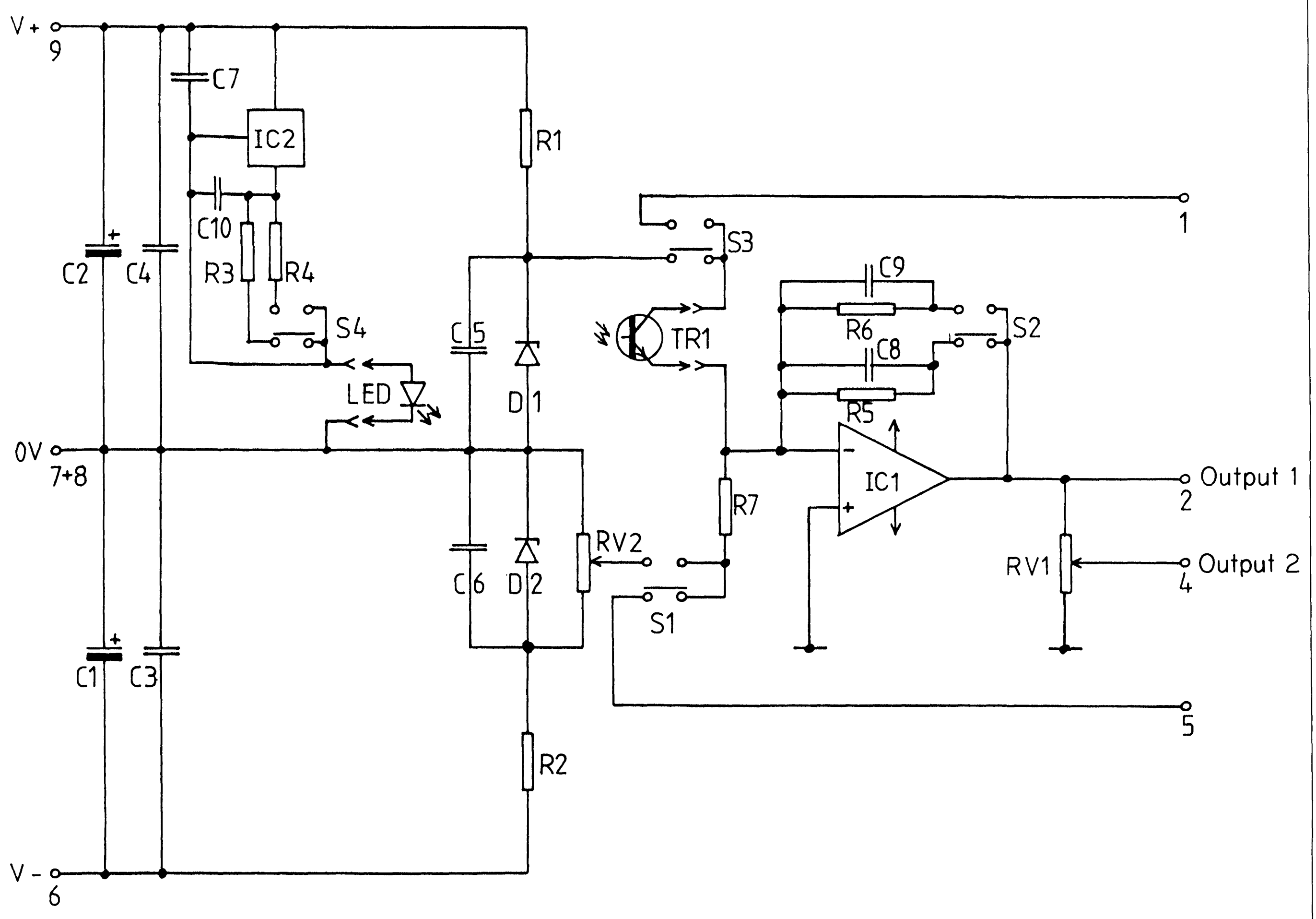

*Figure 1. Circuit diagram of phototransducer. Key to components:*

*Resistors:*

| | | | | | |
|---|---|---|---|---|---|
| *R1* | *4K7* | *R4* | *820R* | *R7* | *2M2* |
| *R2* | *1K* | *R5* | *10M* | *RV1* | *10k* |
| *R3* | *150R* | *R6* | *100M* | *RV2* | *10k* |

*Capacitors:*

| | | | | | |
|---|---|---|---|---|---|
| *C1* | *10μF* | *C4* | *100nF* | *C7* | *220nF* |
| *C2* | *10μF* | *C5* | *100nF* | *C8* | *22nF* |
| *C3* | *100nF* | *C6* | *100nF* | *C9* | *2nF2* |
| | | *C10* | *470nF* | | |

*Semiconductors:*

| | | | | | |
|---|---|---|---|---|---|
| *LED* | *(chosen to suit particular application)* | *D1* | *BZY88C10* | *IC1* | *LF351N* |
| *TR1* | *TIL78* | *D2* | *BZY88C10* | *IC2* | *78L05* |

*Switches:*

*S1–4 four-way DIL switch*

capillary was of uniform diameter and no part of the cell construction impinged upon the flowing stream; and (*b*) that the inert nature of the capillary allowed the cell to be used with corrosive materials and organic solvents. It was found that the fact that the ends of the capillary were bent into a loop in order to fit the housing could have a very slight effect upon the flow pattern, but for applications where this was considered to be a disadvantage (such as dispersion studies) an alternative method of mounting was used. In this arrangement, the encapsulating box was dispensed with, allowing the capillary to remain straight.

A disadvantage of this type of cell was that it was prone to base-line drift, which limited its usable sensitivity. This was eventually overcome by replacing the glass capillary with one made of Kel-F: an extremely inert, clear plastic material. It is not yet known why the Kel-F cells produced less drift than the glass ones, although one possible explanation may be that deposits and tiny air-bubbles present in the system attached themselves more readily to the glass capillary than to the Kel-F, giving rise to a greater amount of base-line noise and drift.

A number of different flowcells, having tubing internal diameters in the range 0·3–2 mm, have been employed successfully with the phototransducer. Those flowcells having a small tubing, internal diameter (i.d.) (0·3 mm), exhibited a poor signal-to-noise ratio, but this is to be expected bearing in mind the extremely short path-length. A conventional longitudinal flowcell of path-length 10 mm has also been used with the phototransducer, with good results.

In a parallel study, it has been shown that the revised phototransducer fitted with a flowcell of tubing i.d. 0·8 mm yields peaks which are sharper than those obtainable from an annular conductivity cell, and avoids contributing to the dispersion of the sample.

### Assessment of performance

The performance of the redesigned phototransducer and flowcell was assessed in an FIA system. Solutions containing lead ($Pb^{2+}$) ion in the range 0–25 ppm were injected into a carrier

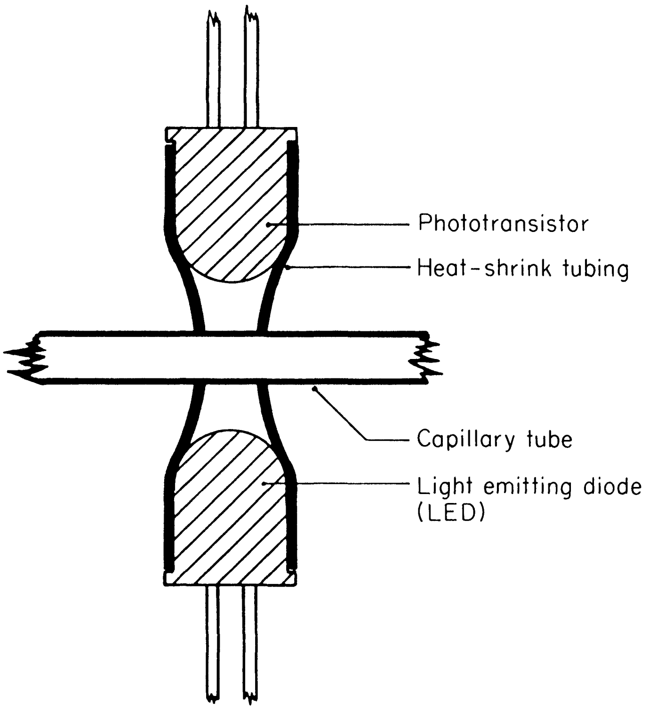

*Figure 2(a). Cross-section through capillary flowcell.*

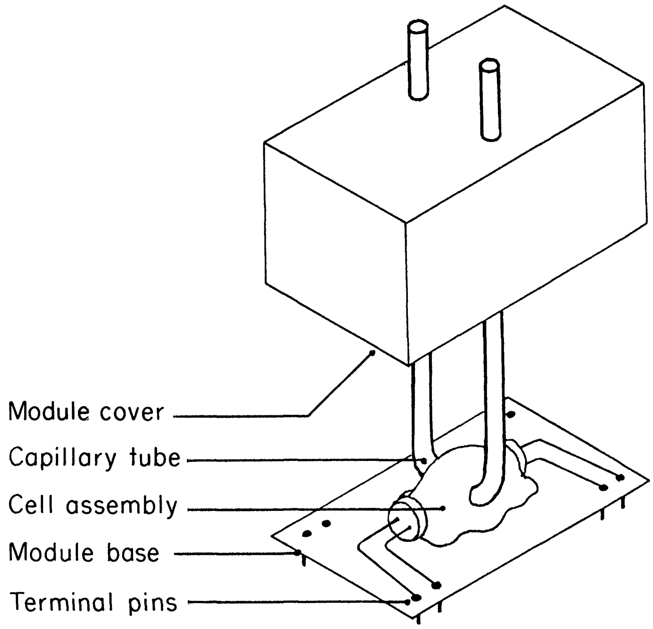

*Figure 2(b). Exploded view of flowcell housing showing method of assembly.*

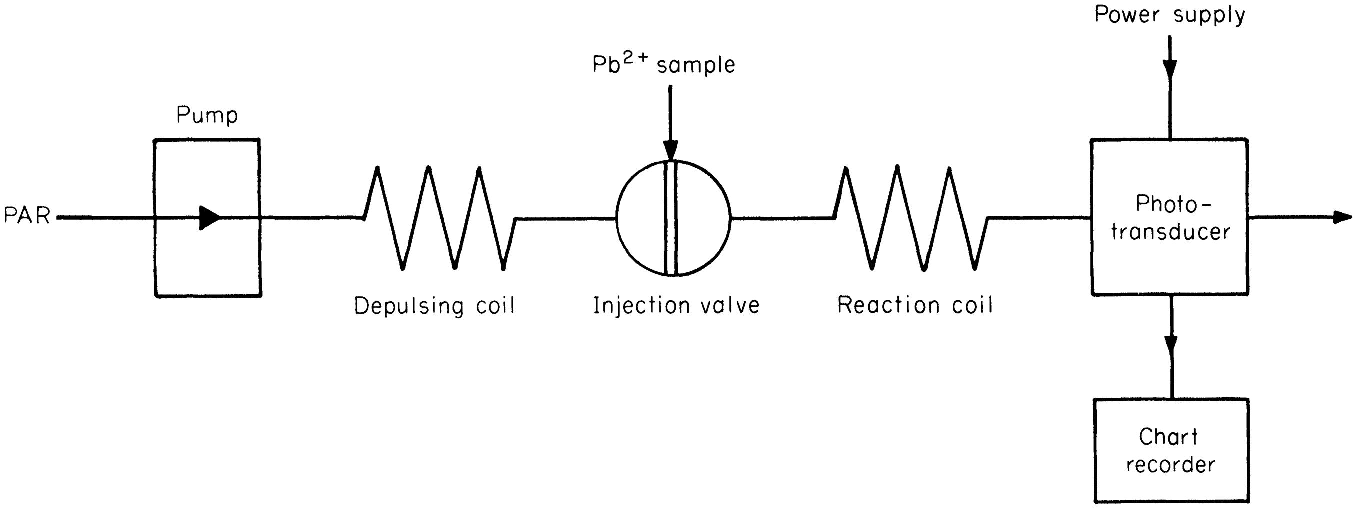

*Figure 3. Schematic layout of apparatus.*

stream of aqueous 4(2-pyridylazo)-resorcinol (PAR) and the resultant coloured species detected using the phototransducer fitted with a flowcell having an LED with a maximum emission at 565 nm.

### Reagents

*Lead stock solution:* an aqueous solution containing 1·000 g/dm$^{-3}$ lead was prepared from anhydrous lead nitrate (Analar) dissolved in dilute (about 10$^{-3}$ mol/dm$^{-3}$) acetic acid to prevent the formation of insoluble lead hydroxide.

*PAR solution:* 10$^{-3}$ mol/dm$^{-3}$ aqueous 4-(2-pyridylazo)-resorcinol (Aldrich).

### Experimental

The FIA system shown in figure 3 was assembled. It consisted of a peristaltic pump (Type 132100, Desaga Gmbh, FR Germany) which pumped the carrier solution at a rate of 3·0 ml/min through a depulsing coil of 0·5 m of silicone tubing of 1·0 mm i.d. and into an injection valve (Teflon valve type 5020, Rheodyne Inc., USA) equipped with a sample loop of 0·25 ml capacity. The carrier passed into a reaction coil of length 0·40 m and thence into the phototransducer. With the exception of the pump tubing and depulsing coil, all other tubing in the system was PTFE (i.d. 0·8 mm).

A number of samples in the range 0–25 ppm were prepared

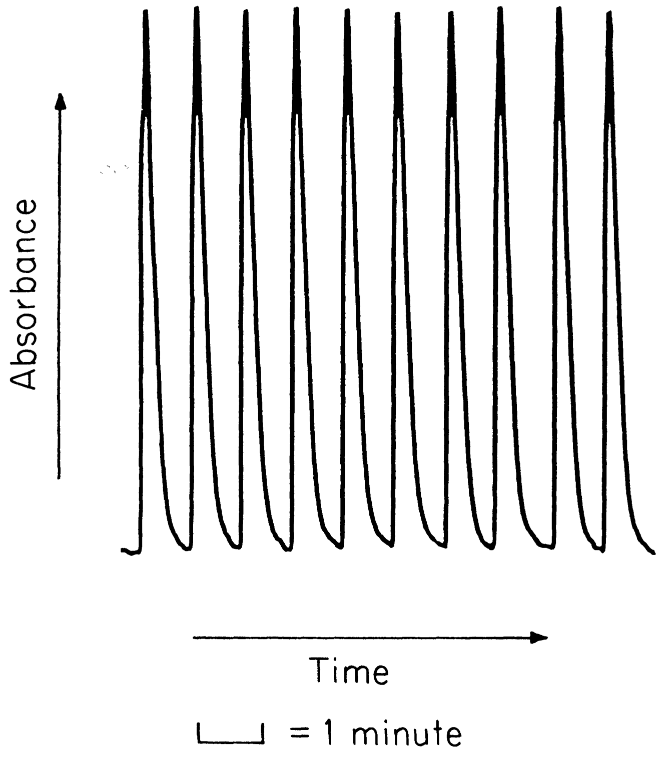

*Figure 4. Recorder trace obtained for a series of 10 samples, each containing 25 ppm $Pb^{2+}$.*

from the stock solution. These were injected into the carrier stream and the resultant output from the phototransducer was monitored by means of a chart recorder (type B5117-5D, Gallenkamp Ltd). A graph of peak height versus concentration was obtained. In addition, a series of 10 samples each containing 25 ppm $Pb^{2+}$ was injected into the system. The heights of the resultant peaks were examined and the relative standard deviation determined.

### Results and discussion

The calibration curve obtained is a straight line of the form

$$H = 3{\cdot}67C - 6{\cdot}86$$

where $H$ is the peak height in mm and $C$ is the sample concentration in ppm. The correlation coefficient is 0·9997.

Figure 4 shows the peaks obtained by injecting a series of 10 samples each of 25 ppm $Pb^{2+}$. Statistical analysis of the results gave a mean peak height of 84·15 mm, a standard deviation of 0·48 mm and a relative standard deviation of 0·58%.

More than 15 phototransducers have been constructed to date, and the results obtained above are typical of any of the units. A number of the units have found application in the development of new analytical techniques based upon FIA, whilst their low cost (about £25) and robust construction make them eminently suitable for use in teaching undergraduate students the principles of FIA, withstanding a considerable amount of misuse and rough handling with no ill effects.

The limitation of the phototransducer lies in the limited number of wavelengths for which LEDs are available: at present the only readily available wavelengths in the visible range are green (565 nm), yellow (590 nm) and red (625 nm). In practice this has been found to present less of a problem than might at first appear, since it is frequently possible to monitor the absorbance of a particular species at a wavelength other than that of maximum absorbtivity. In a recent example, the phototransducer was used in the development of an analytical method based on FIA for a drug which underwent reaction to form a compound with an absorbance maximum at approximately 490 nm. By using a flowcell incorporating a green LED (565 nm), perfectly adequate sensitivity was obtained.

Alternatively, where the wavelength of maximum absorbance for the product lies too far from the LED wavelength for a useable sensitivity to be obtained, one may instead monitor the decrease in absorbance of the reagent and relate this to the original sample concentration.

### Conclusion

The improved phototransducer provides a robust low-cost photometric detector suitable for use in FIA and other continuous-flow analytical techniques, on a wide range of different chemical systems. Where identical flowcells are used, the new circuit provides improved sensitivity compared with the original design.

Future work to improve the performance of the unit will concentrate upon interfacing the phototransducer to a microcomputer, with the aim of improving the signal-to-noise ratio of the unit through the use of Fast Fourier Transform (FFT) and digital filtering techniques.

### Acknowledgements

The authors are grateful to the Department of Industry for the provision of a studentship for T.J.S.

### References

1. Betteridge, D., Dagless, E. L., Fields, B., and Graves, N. F., *Analyst*, **103** (1978), p. 897.
2. Goad, T. B., Ph.D. thesis, University of Wales (1982).

